# Maternal and Fetal Outcome in Pregnant Women With Critical COVID-19 Treated With Tocilizumab in a Tertiary Care Hospital in Dubai

**DOI:** 10.7759/cureus.34395

**Published:** 2023-01-30

**Authors:** Bindu Isaac, Komal Hazari, Deemah K Harb, Ayaz K Mallick, Widad Abdelkareem, Abeir Ammar, Taghrid Gergawi, Eiman Saeed Al Zahmi, Amar H Khamis

**Affiliations:** 1 Obstetrics and Gynecology, Latifa Women and Children Hospital, Dubai Health Authority, Dubai, ARE; 2 Internal Medicine, Latifa Women and Children Hospital, Dubai Health Authority, Dubai, ARE; 3 Clinical Biochemistry, College of Medicine, King Khalid University, Abha, SAU; 4 College of Medicine, Mohammed Bin Rashid University of Medicine and Health Sciences, Dubai, ARE

**Keywords:** covid-19, maternal and fetal outcome, cytokine storm, tociluzumab, pregnancy

## Abstract

Introduction: Pregnancy, complicated by coronavirus disease 2019 (COVID-19), results in higher hospitalization and mortality rate. Pathogenesis of COVID-19 is similar to any other systemic inflammatory condition but results in a cytokine storm of higher magnitude causing severe acute respiratory distress syndrome and multiorgan failure. Tocilizumab, a humanized monoclonal antibody, targets soluble and membrane-bound IL-6 receptors and is used in the treatment of juvenile idiopathic arthritis, rheumatoid arthritis, and cytokine release syndrome. However, studies exploring its role in pregnancy are minimal. Hence, this study was done to study the effect of tocilizumab on maternal and fetal outcomes in critical COVID-19 pregnant women.

Methodology: A retrospective study was conducted on 28 pregnant women with critical COVID-19 who received tocilizumab. Clinical status, chest x-ray, biochemical parameters, and fetal well-being were monitored and documented. The discharged patients were followed up through telemedicine.

Result: On treatment with tocilizumab, improvement was seen in the number of zones and patterns of chest x-ray, along with 80% reduction in the c-reactive protein (CRP) levels. Based on the WHO clinical progression scale, 20 patients improved by the end of first week, and by the end of first month, 26 patients became asymptomatic. Two patients died during the course of the disease. No fetal adverse effects were noted.

Conclusion: Based on the encouraging response and as tocilizumab did not impart any adverse effects on the pregnancy, tocilizumab may be administered as an adjuvant to critical COVID-19 pregnant women in their second and third trimesters.

## Introduction

Globally, as of September 2022, there have been more than 610 million confirmed cases of coronavirus disease 2019 (COVID-19), including more than six million deaths, reported to WHO [1]. The UAE has seen over one million COVID cases with 2342 deaths, with pregnant women forming a considerable number of cases [2]. Compared to non-pregnant women, pregnant women were affected more severely with higher intensive care unit (ICU) admissions, invasive ventilation, extracorporeal membrane oxygenation, and death [3]. Published data in 2021 showed a 3.5-fold higher hospitalization rate and 13.6-fold higher mortality among COVID-19 complicated pregnancies than in non-pregnant COVID cases within the same age group [4].

Numerous studies have linked the severity of COVID-19 to cytokine storm, a rapid systemic inflammatory response to infections that results in increased immune cell activation and release of pro-inflammatory cytokines, causing multiorgan dysfunction [5]. Tocilizumab (TCZ) is a humanized recombinant monoclonal antibody directed against the human IL-6 receptor that inhibits IL-6 from binding to its receptor and exerting immunosuppressive effects. Since IL-6 is one of the key cytokines involved in an infection-induced cytokine storm, treating COVID-19 with tocilizumab is effective, especially in critically ill patients, as shown in the randomized, embedded, multifactorial, adaptive platform trial for community-acquired pneumonia (REMAP-CAP) trial completed in 2021, which is one of the largest trials (n=803) on tocilizumab [6].

Tocilizumab currently is approved by the United States Food and Drug Administration (US FDA) for the treatment of severe cytokine release syndrome (CRS), idiopathic arthritis, rheumatoid arthritis, and giant cell arteritis [7]. Presently, the FDA has categorized tocilizumab as a category C drug in pregnancy [8]. A review compiled data from 610 pregnant women on tocilizumab reported that tocilizumab use in pregnancy had its limitations and the data were not sufficient to identify the entire spectrum of potential adverse outcomes [7]. Tocilizumab has been used in pregnant women with rheumatoid arthritis with relatively safe fetus outcomes. The aim of this study was to study the effect of tocilizumab on maternal and fetal outcomes in critical COVID-19 pregnant women.

## Materials and methods

Study design

We conducted a descriptive retrospective study of pregnant women infected with COVID-19 and treated with tocilizumab from January 2021 to June 2021 at Latifa Women and Children Hospital, Dubai, UAE, one of the main tertiary care centres specializing in maternal and neonatal services.

Sample selection and data collection

The medical records of all pregnant women with confirmed COVID-19 treated with tocilizumab were included in the study. Pregnant women on other immunosuppressant drugs like azathioprine, adalimumab, and sulfasalazine were excluded from the study.

The National Institute of Health (NIH) criteria were applied to categorize the clinical spectrum of patients with COVID-19 (Appendix 1). Radiologically, the lungs were divided into six zones for chest x-ray severity scoring, three on each side, upper, middle, and lower [9]. Opacities were classified into reticular, ground glass, patchy, and dense consolidation patterns. The WHO clinical progression scale was implemented to monitor the clinical outcome [10]. All the admitted pregnant patients with COVID-19 received the standardized treatment as per the UAE national guideline for the management of COVID-19 illness, which was implemented in January 2021 (Appendix 2).

Data was collected regarding demographic characteristics, past medical and obstetric history, concurrent medication, and gestational age. Laboratory parameters, radiological testing, vital signs and symptoms, and microbiological tests were regularly monitored according to the clinical condition of the patient since time of admission. Tocilizumab therapy was started based on any of the following criteria: (1) clinical worsening with increasing oxygen requirements or WHO score of 6 and above. (2) Progressively worsening radiological findings. (3) Worsening inflammatory markers including IL-6, c-reactive protein (CRP), D-dimer, and ferritin levels. Women with serum liver enzymes (aspartate transaminase {AST} and alanine transaminase {ALT}) elevated more than five times the upper normal limits were excluded from the study.

Systemic corticosteroids were given as a part of protocol to all patients who were admitted as critical COVID. Twenty-eight patients who fulfilled the criteria were given two doses of 400 mg of tocilizumab intravenously 24 hours apart. After administration of tocilizumab, patients were evaluated on days 2, 5, 10, 14, and 28. An improvement of one point on the WHO clinical progression scale was considered as significant.

Maternal and fetal outcomes including gestational age and mode of delivery, the neonatal SARS-CoV-2 results, somatometric evaluation including weight, length, head circumference, APGAR scores, neonatal ICU admission, and follow-up details were also collected from the records. Congenital anomalies in any patient were identified and documented. The discharged patients were followed up through telemedicine on day 14, day 28, and day 60 from their admission.

Statistical analysis

Data were entered into the computer using IBM-SPSS for Windows version 28.0 (Chicago, IL: SPSS Inc.). Measure of tendency and dispersion were used to describe the continuous data, while categorical variables were described by using frequencies and proportions. Rate of change was performed between, before, and after treatment.

## Results

During the study period, 28 pregnant patients received tocilizumab. The mean gestational age was 30.11 (±5.73) weeks. The mean maternal age was 33.21 (±5.94) years, ranging between 22 and 44 years, and mean BMI was 36.96 (±16.83) (Table 1).

**Table 1:** The age, obstetrical data, and ethnicity of pregnant women with COVID-19 (n=28). COVID-19: coronavirus disease 2019

Patient demographics	Mean (SD)
Age (years)	33.21 (5.94)
BMI	36.96 (16.83)
Parity	1.32 (1.72)
Gestational age (in weeks)	30.11 (5.73)
Ethnicity	Number of patients (%)
Asia	14 (50)
Arab	12 (42.8)
European	1 (3.5)
Africa	1 (3.5)

The most common comorbidity reported was gestational diabetes mellitus (GDM) in 32% (9/28), followed by hypothyroidism in 10.7% (3/28). The mean days from COVID-19 symptoms onset to admission was around 8.7±3 days. Tocilizumab was started around the fourth post-hospitalization day. The mean duration of hospital stay was 13.5 (±19.96) days. All the patients were transferred to the ICU, with an average ICU stay of 12.2 (SD 18.96) days (Table 2).

**Table 2:** A consolidated table showing the hospital course, comorbidities, concurrent treatment, maternal outcome. ICU: intensive care unit; GDM: gestational diabetes mellitus

Hospital course	Mean days (SD)
Days from the onset of COVID-19 symptoms to admission	8.7 (3)
Day of starting tocilizumab following admission	3.96 (3.47)
Duration of stay in the ICU	12.26 (18.96)
Risk factor (n=28)	Number of patients (%)
Nil	16 (57.14)
GDM	9 (32.1)
Hypothyroid	3 (10.7)
Concurrent treatment	Number of patients (%)
Steroid	28 (100)
Kaletra	3 (10.7)
Bioferon	28 (100)
Remdesivir	24 (85.7)
Antibiotics	28 (100)
Plasma	2 (7.1)
Follow-up at 28 days (n=28)	Number of patients (%)
Discharged	25 (90%)
ICU	2 (7.2%)
Mortality	1 (3.6%)
Follow-up at 60 days (n=28)	Number of patients (%)
Discharged	25 (90%)
ICU	1 (3.6%)
Mortality	2 (7.2%)

Following admission, chest x-rays were taken before the initiation of treatment, and follow-up was done once the treatment started. As shown in Table 3, pre-treatment 75% (21/28) of the patients had an x-ray score of four zones involved, of which 11/28 had patchy opacities and 9/28 had consolidation pattern; 7% (2/28) had five zones involved, of which 1/28 had patchy and 1/28 had consolidation pattern; 14% (4/28) had six zones involvement, of which 2/28 had patchy and 2/28 had consolidation patterns. Analysis of the x-ray pattern showed that 50% (14/28) had patchy distribution, while 46% (13/28) had consolidations. Repeat chest x-ray 48 hours post-treatment showed 14% (4/28) had two zones involvement, 28% (8/28) had three zones involvement, 46% (13/28) had four zones involvement, and 14% (4/28) continued to have six zones involvement. It was observed that patchy opacities were the most common pattern seen in 39% (11/28) of the x-rays, followed by reticular pattern in 36% (10/28), consolidation in 14% (4/28), ground glass opacities in 10% (4/28).

**Table 3:** The zones and x-ray pattern before and after treatment (n=28).

Zones	Pre-tocilizumab treatment				48 hours post-tocilizumab treatment			
	Reticular	Ground-glass	Patchy	Consolidation	Reticular	Ground-glass	Patchy	Consolidation
2	0	0	0	0	3	0	1	0
3	0	0	0	1	3	0	5	0
4	0	1	11	9	4	4	3	2
5	0	0	1	1	0	0	0	0
6	0	0	2	2	0	0	2	2
Total	0	1	14	13	10	4	11	4

The biochemical parameters of the patients before and after starting the treatment are summarized in Table 4. Clinically significant findings included a 73.5% increase in serum IL-6, 37.6% increase in serum ferritin, 50.6% increase in serum glutamic pyruvic transaminase (SGPT), 23.3% increase in absolute lymphocyte count, and 8.9% increase in serum lactate dehydrogenase (LDH) along with a 78.8% decrease in CRP and 24.2% decrease in serum glutamic-oxaloacetic transaminase (SGOT) after starting the treatment. The D-dimer levels were comparable both before and after treatment.

**Table 4:** The mean (SD) levels of various biochemical profiles before and after treatment. *Rate of change was performed between before and after treatment. CRP: c-reactive protein; LDH: lactate dehydrogenase; SGOT: serum glutamic-oxaloacetic transaminase; SGPT: serum glutamic pyruvic transaminase; IL-6: interleukin 6

Laboratory parameter	During admission	Before treatment	After treatment (day 2)	Rate of change*
Absolute lymphocyte count (1.0-3.0×10^3^ cells/µL)	0.88 (0.41)	0.90 (0.44)	1.11 (0.63)	23.3%
CRP (0-7 mg/L)	56.9 (34.5)	55.11 (41.9)	11.7 (19.4)	-78.8%
IL6 (<7.0 pg/mL)	42.1 (27.4)	214.2 (681)	371.7 (448.9)	73.5%
Ferritin (10-150 ng/mL)	428.1 (840.4)	638.5 (676.4)	878.8 (1336.6)	37.6%
LDH (105-222 U/L)	249 (106.3)	326.7 (158.7)	355.7 (192.6)	8.9%
D-Dimer (<0.5 mcg/mL FEU)	1.99 (2.8)	1.33 (1.28)	2.01(1.26)	0.51%
SGOT (5-40 IU/L)	-	64.1 (71.9)	48.6 (71.9)	-24.2%
SGPT (5-40 IU/L)	-	42.9 (38.5)	64.6 (51.5)	50.6%

As shown in Table 5, all the patients had a WHO score of six before starting the treatment with tocilizumab. By the second post-tocilizumab day, one patient had a WHO severity score of five. By the fifth and 10th day, another eight and three patients improved their severity score to five, respectively, eight and five patients improved their score to four, but by day 10, eight patients continued to have a severity score of 6, whereas 11 patients showed an improvement to the WHO severity score of two. At the end of the fourth week, 85% (24/28) were discharged, 11% (3/28) remained in the ICU, and one patient died on the 21st post-tocilizumab administered day. By the end of the second month, another one patient was discharged, whereas one patient continued to be critical in the ICU, and another patient died on the 42nd post-treatment day. Two patients who remained on the ventilator for more than 20 days showed positive microbiological culture; one patient had Pseudomonas in the blood and respiratory culture and the second patient had fungi and multidrug-resistant bacteria in the blood and respiratory culture. On the ventilator for a long time, one critical patient developed massive axonal neuropathy of tibial, peroneal, and sural nerve post-COVID and underwent physiotherapy with good recovery. Four patients developed pre-eclampsia during COVID treatment and were managed according to hospital protocol.

**Table 5:** WHO severity score of the pregnant COVID-19 women pre-tocilizumab and followed up on days 2, 5, 10, 30, and 60. COVID-19: coronavirus disease 2019

WHO severity scale	Admission	Pre-tocilizumab	Post-tocilizumab administration day				
			Day 2	Day 5	Day 10	Day 30	Day 60
1	-	-	-	-	1	24	25
2	-	-	-	-	11	-	-
3	1	-	-	-	-	-	-
4	3	-	-	8	5	-	-
5	24	-	1	8	3	-	-
6	-	28	27	12	8	1	-
7	-	-	-	-	-	-	-
8	-	-	-	-	-	2	1
9	-	-	-	-	-	-	-
10	-	-	-	-	-	1	2

The perinatal and fetal outcomes of the patients are summarized in Table 6. Following discharge, five patients were lost on follow-up. There were nine preterm deliveries, out of which seven were delivered due to worsening maternal conditions secondary to COVID-19, one (of 23) each due to abruptio placenta and fetal distress. Cesarean sections were performed for 18 of 23 patients, indications being eight cases of worsening maternal COVID condition, six cases of prolonged labor, and one case each of abruption, intrauterine growth restriction (IUGR), fetal distress, and pre-eclampsia. All the babies reported negative for COVID-19 at birth. Sixty percent of the newborns had an APGAR score of eight and nine at the 1st and 5th minute, respectively. Ten out of 23 babies were admitted to neonatal intensive care unit out of which nine were due to prematurity and one baby was admitted due to respiratory distress. Septic workup of the babies admitted to the neonatal intensive care unit was negative for all the babies. The somatometric measurements which include height, weight, head, and chest circumference for all the newborns were within normal limits. Out of 23 babies delivered, 14 babies had normal development at follow-up examination at one year of age, four babies had normal follow-up till six months of age, and five babies were lost to follow-up after discharge.

**Table 6:** Perinatal and fetal outcome. NICU: neonatal intensive care unit; LSCS: lower segment cesarean section

Mode of delivery (n=23)	Number of patients (%)
Normal vaginal delivery	5 (21.7)
LSCS	18 (78)
Number of term babies	14 (61)
Number of preterm babies	9 (39)
Preterm deliveries due to maternal worsening condition due to COVID-19 (n=9)	7 (78)
Preterm deliveries due to obstetric condition (n=9)	2 (22)
NICU admissions	10 (43.7)
APGAR score	Number of patients (%)
APGAR score 7 and above at 1 min	22 (95.6)
APGAR score 7 and above at 5 min	23 (100)

## Discussion

COVID-19 is an air-borne disease primarily affecting the respiratory system and inducing a severe inflammatory response, thereby releasing numerous inflammatory mediators such as IL-1, IL-6, and tumor necrosis factor-alpha (TNF-α) leading to cytokine storm which has a detrimental effect [11]. Controlling the cytokine storm early on with immunomodulatory treatments is critical for increasing treatment success and decreasing mortality in patients with COVID-19 [12]. COVID-19-infected patients showed better clinical outcomes and higher survival rates with intravenously administered tocilizumab [13,14]. Pregnant women with viral infections generally are more vulnerable to developing severe diseases than non-pregnant women and should be managed as a high-risk case [15,16]. Currently, the data regarding the use and safety of tocilizumab in pregnancy is limited. However, some studies have reported that tocilizumab does not cross the placenta or, even if it does, the amount is minimal. Also, no congenital anomalies are reported with tocilizumab [17]. Saito et al. concluded that tocilizumab was safe during pregnancy with low transplacental transmission, low amount in breast milk, and no detectable absorption by the newborn [18].

Pregnant women were categorized based on the WHO severity scale following admission (Appendix 3). The WHO severity scale has been proposed by the WHO Working Group on the Clinical Characterization and Management of COVID-19 infection to monitor the patient's progression. Tocilizumab therapy was initiated when our patients deteriorated clinically and radiologically with WHO score worsening from baseline of four and five on admission to a score six on approximately day four.

After initiating the treatment, gradual clinical and radiological improvement was observed in the majority of our patients. No major maternal or fetal adverse effects were observed. Similar findings were reported by Anghel et al., where statistically significant improvement was observed in consolidating lesions in x-rays of 79 COVID-19 patients seven to 10 days after starting treatment with tocilizumab [19]. Although empirical antibiotics were started to avoid the potential risk of superadded infections, they were discontinued upon negative cultures except for one patient who remained on the mechanical ventilator and showed the presence of *Candida glabrata *(respiratory tract), *Klebsiella pneumonia*, carbapenem-resistant pseudomonas (trachea aspirate), and extended-spectrum beta-lactamase (ESBL) positive Klebsiella (urine) in culture.

As per the biochemical tests, our patients remained in a hyperinflammatory state before tocilizumab. However, two days after starting tocilizumab, CRP showed a reduction by 78.8%, whereas other markers continued to remain elevated. This is because markers such as ferritin and LDH have a longer half-life and remain elevated for up to 48 hours [20,21]. CRP has a shorter half-life of 19 hours; therefore, rapid reduction was seen by the second day [22]. The increase in serum IL-6 is a temporary effect seen because of the IL-6 receptor-blocking action of tocilizumab hence increasing its circulatory levels [23].

Two patients died during the course of treatment, one at the end of first month of admission to hospital and second at the end of second month, rest of them were stable and discharged home. In a systemic review by Debrabandere et al., it was concluded that 68.9% of the women delivered by cesarean section due to complications from COVID-19 [24,25]. Similarly, in our study, 78% of pregnant women delivered by emergency lower segment cesarean section (LSCS). Among these, 50% were due to complications by COVID-19. None of the newborns tested positive for COVID-19, and no anomalies were detected in any infant.

Study limitations

Limitations included the retrospective nature of this study, the lack of control group, short follow-up period, and the small sample size. Another limitation was the absence of a control group which could have eliminated the confounding effect of other antiviral drugs on the prognosis of the patients. A causative link between tocilizumab and congenital anomalies cannot be completely ruled out as our study cohorts were in the second and third trimesters with no exposure to the drug during the first trimester, which is the period of organogenesis.

## Conclusions

In this retrospective study, Tocilizumab, an anti-interleukin-6 receptor antibody approved for multiple inflammatory diseases, was administered to pregnant women with critical COVID-19 illness to study its effectiveness in reducing mortality and the requirement of mechanical ventilation. In our study, the WHO severity scale was used to measure clinical improvement. On day 10, 20/28 patients showed significant clinical improvement and were on their way to recovery; the average length of ICU stay was 12 days, while only 2/28 patients required mechanical ventilation. Out of the 23 women who delivered in our institution, no anomaly at birth or infections were reported in the newborns. Tocilizumab may be considered an adjuvant treatment for pregnant women with critical COVID-19 illness in their second and third trimesters. However, more studies with a larger study group may be required to substantiate our conclusion further.

## Figures and Tables

**Figure 1:**
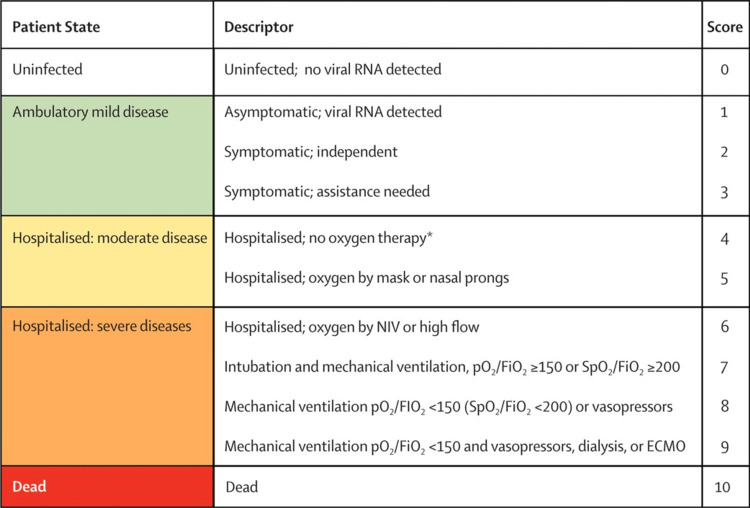
WHO clinical progression scale.

## Appendices

 Appendix 1

**Table 7:** National Institute of Health COVID-19 disease severity criteria. COVID-19: coronavirus disease 2019

Asymptomatic/pre-symptomatic infection	Individuals who test positive for SARS-CoV-2 using a virologic test (i.e., a nucleic acid amplification test {NAAT} or an antigen test) but who have no symptoms that are consistent with COVID-19
Mild illness	Individuals who have any of the various signs and symptoms of COVID-19 (e.g., fever, cough, sore throat, malaise, headache, muscle pain, nausea, vomiting, diarrhea, loss of taste and smell) but who do not have shortness of breath, dyspnea, or abnormal chest imaging
Moderate illness	Individuals who show evidence of lower respiratory disease during clinical assessment or imaging and who have an oxygen saturation measured by pulse oximetry (SpO_2_) ≥94% on room air at sea level
Severe illness	Individuals who have SpO_2_ <94% on room air at sea level, a ratio of arterial partial pressure of oxygen to fraction of inspired oxygen (PaO_2_/FiO_2_) <300 mmHg, a respiratory rate >30 breaths/min, or lung infiltrates >50%
Critical illness	Individuals who have respiratory failure, septic shock, and/or multiple organ dysfunction

Appendix 2

**Table 8:** Standardized treatment regimens for COVID-19. COVID-19: coronavirus disease 2019

Generic name	Dose	Duration
Hydroxychloroquine	Two loading doses of 400 mg on day one followed by 200 mg twice daily	7-14 days
Chloroquine base	300 mg (base) twice daily	5-7 days
Lopinavir-Ritonavir (200 mg/50 mg)	400 mg/100 mg twice daily	5-7 days (maximum 14 days)
Remdesivir	200 mg IV on day 1, followed by 100 mg IV	Total duration 5 days
Tocilizumab	4-8 mg/kg (max 400 mg) followed by a second dose after 8-12 hours.	2 doses within 24 hours
Pegylated interferon nebulized interferon	180 mcg, maximum of 2 doses 1 week apart 5 million units BD	2 doses within one week 5-10 days
Low molecular weight heparin (LMWH) (prophylactic)	According to body weight: 50-90 kg: 40 mg once daily, 91-130 kg: 60 mg once daily, and 131-170 kg: 80 mg once daily.	Till clinical improvement
Low molecular weight heparin (LMWH)-therapeutic in critical COVID-19 pneumonia patients	50-90 kg: 40 mg twice daily 91-130 kg: 60 mg twice daily 131-170 kg: 80 mg twice daily	Till clinical improvement
Methylprednisolone	0.5-1 mg/kg in 2 divided doses	3 days in non-ICU and 5-7 days in ICU patients.
N-acetyl cysteine	PO 400 mg thrice daily nebulization 200-400 mg thrice daily	5-7 days

Appendix 3 
